# How to Put Wellness on the Prescription Pad: Recommendations

**DOI:** 10.3747/co.v15i0.277

**Published:** 2008-08

**Authors:** P.R. Fortin

**Affiliations:** Toronto Western Hospital, Toronto, Ontario, Canada

**Keywords:** Integrative oncology, holistic cancer treatment

## Abstract

The Integrating Wellness into Cancer Care conference was held at the University of Toronto, October 4–5, 2007, and was dedicated to the memory of the late Dr. Véronique Benk. This article summarizes the workshops at that conference.

The notion of wellness and an integrated approach should be introduced from the outset as part of the cancer patient’s management. Having wellness as part of the treatment sets a standard for taking care of the patient’s emotional, spiritual, physical, and nutritional needs, and for providing information on complementary therapies. A focus on holistic supportive care during treatment and survivorship is important.

The whole medical team should support an integrative program. Referral to an education program and one-to-one assessments by a point person such as an advanced nurse practitioner, a social worker, or a psychological counsellor with appropriate special training should be mandatory. The concept of a pathfinder or cancer guide was discussed.

